# Nanopore Technology and Its Applications in Gene Sequencing

**DOI:** 10.3390/bios11070214

**Published:** 2021-06-30

**Authors:** Bo Lin, Jianan Hui, Hongju Mao

**Affiliations:** 1 State Key Laboratory of Transducer Technology, Shanghai Institute of Microsystem and Information Technology, Chinese Academy of Sciences, Shanghai 200050, China; linbo201@mails.ucas.ac.cn (B.L.); jiananhui2@mail.sim.ac.cn (J.H.); 2 Center of Materials Science and Optoelectronics Engineering, University of Chinese Academy of Sciences, Beijing 100049, China

**Keywords:** nanopore technology, nanopore sequencing, diagnosis of cancer, biosensor

## Abstract

In recent years, nanopore technology has become increasingly important in the field of life science and biomedical research. By embedding a nano-scale hole in a thin membrane and measuring the electrochemical signal, nanopore technology can be used to investigate the nucleic acids and other biomacromolecules. One of the most successful applications of nanopore technology, the Oxford Nanopore Technology, marks the beginning of the fourth generation of gene sequencing technology. In this review, the operational principle and the technology for signal processing of the nanopore gene sequencing are documented. Moreover, this review focuses on the applications using nanopore gene sequencing technology, including the diagnosis of cancer, detection of viruses and other microbes, and the assembly of genomes. These applications show that nanopore technology is promising in the field of biological and biomedical sensing.

## 1. Introduction

Nanopore technology refers to nano-scale holes embedded in a thin membrane structure to detect the potential change when charged biological molecules smaller than nanopore pass through the hole [1]. Therefore, nanopore technology has the potential to sense and analyze single-molecule amino acid, DNA, RNA, etc. [2,3]. In this review, we will focus on the applications of nanopore technology in gene sequencing.

Nucleic acid is an important genetic material for most of the living body, and accurate sequencing of the nucleic acids is important for biomedical research, which would be useful for diagnosing human diseases and providing personalized medicine [4]. Since the last century, gene sequencing technology has developed dramatically, and now the nanopore technology has taken a leading role in the area of gene sequencing.

**Figure 1:**
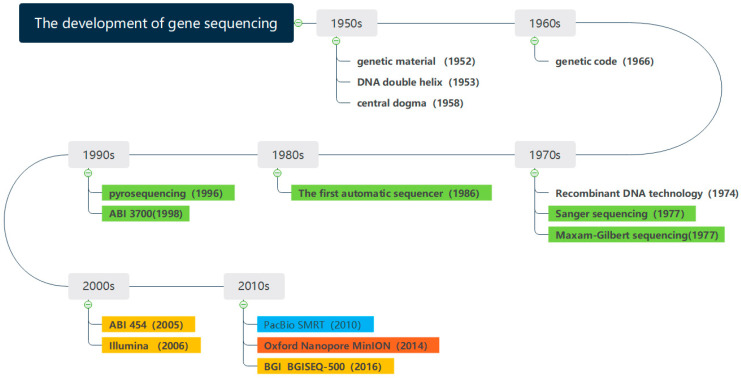
The development of gene sequencing technology.

Figure 1 shows the development of gene sequencing technology. Since the 1950s, the determination of DNA double helix structure has preluded the research of genes. With the development of sequencing technology, the milestones have been made in gene sequencing. The whole genome of phage φ174 DNA was sequenced in 1977 [5], which marked the completion of the first genome sequencing in history. The Human Genome Project [6], proposed in 1990, laid a solid foundation for the further study of the human genome. The sequencing of Saccharomyces cerevisiae was completed in 1996, which completed the eukaryotic genome for the first time. The gene sequencing technology has been constantly innovating and evolving in the direction of lower cost, higher throughput, and faster speed [7,8,9,10,11,12,13].

The first-generation sequencing technology is represented by Sanger sequencing, which mainly utilizes chain-terminating method and gel electrophoresis technology. However, the downside of the Sanger sequencing equipment was costly and time consuming. As the Human Genome Project cost $3 billion and took 13 years to complete [6], further application of first-generation gene sequencing technology was limited.

The second- and third-generation sequencing technologies are characterized by high throughput and are also known as Next Generation Sequencing Technologies (NGS) [14]. Roche’s 454 Sequencing Platform [15] and Illumina’s Solexa Sequencing System [16] are the representative. The BGISEQ-500 [17], a second-generation sequencing platform later developed by China’s BGI, has also taken some domestic market shares. Among the third-generation sequencing platforms, PacBio’s SMRT technology has become the backbone with its high throughput and high accuracy.

The fourth generation of gene sequencing technology was the combination between gene engineering technology and computer-aided technology. Oxford Nanopore sequencing technology (ONT) innovatively used the nanopore technology to determine the base sequences from the current distortion when DNA passing through the nanopore [18]. ONT has become one of the most powerful sequencing technologies since its born. It has the advantages of the whole-genome sequencing and disease diagnosis with fast speed and cost-effective performance. Using ONT, the sequencing length of the largest DNA strand is continuously increasing with reported read length of 1 M base pairs [19]. Table 1 shows the comparison of four kind sequencing devices of different sequencing generation.

There are several kinds of nanopore sequencing platforms, including ONT, NabSys, and Sequenom [20]. ONT uses biological nanopore to get the sequence of DNA or RNA. Different from ONT, NabSys uses solid-state nanopore to sequence and it can also reconstruct the DNA map by using the short DNA probe. As for nanopore sequencing technology of Sequenom, it uses a readout system technology for single DNA molecules based on simultaneous optical probing of multiple nanopore. This paper mainly takes ONT as an example to introduce the principle of nanopore sequencing and its applications.

## 2. Principle of Nanopore Technology

Nanopore-based technologies originated from the Coulter and ion channels [24], which could be traced back to the 1990s [1]. Nanopore technology is done by applying a cathode and anode to the solution on the forward and reverse side of the membrane, respectively. Negatively charged biomolecules, such as DNA, can be placed on the forward side and these molecules can pass through pores in the membrane under the electrophoretic force with applied voltage. When different molecules translocate through the pores, the current level can be captured and be further used for calculation in computer-aided tools.

The category of nanopore used in nanopore technology can be divided into two parts, solid-state nanopore and biological nanopore. We first provide a brief overview of these nanopores in the following part.

### 2.1. Solid-State Nanopore

Solid-state nanopore can be fabricated using different methods, such as controlled breakdown, electrochemical reactions, laser etching and laser-assisted controlled breakdown [25]. It breaks the limit of natural occurring nanopores and has many advantages, such as very high chemical stability, control of diameter and channel length, adjustable surface properties, and the potential for integration into devices and arrays [26,27].

Si_3_N_4_ and SiO_2_ nanopores are one of the most widely used nanopores and their fabrication is compatible with the complementary metal oxide semiconductor industrial integrated circuit processes [28]. These nanopores can be ion etched in free-standing Si_3_N_4_ and SiO_2_ films, using argon ion beam or electron beam [29].

### 2.2. Biological Nanopore

Biological nanopore comes from natural protein molecules or artificial nanopores generated by genetic engineering [30]. However, the biological nanopores are frail with features such as short lifetime, intrinsic instability, and strict requirement of a specific environment, which are not able to support a biosensor’s long-term operations.

Biological nanopores are usually produced by selected bacteria, such as α-hemolysin pore protein [31], MspA from Mycobacterium smegmatis [32], and Phi29 from Bacillus subtilis [33]. These biological nanopores are currently used for disease diagnosis [34], gene sequencing [35], and protein sequencing [36].

## 3. Nanopore Sequencing Technology

ONT is a single molecule sequencing technology based on nanopores. The first prototype, MinION, was released in 2014 [37]. The updated platform, PromethION, was released in 2015 with improved throughput. Two versions of PromethION, naming ProtheION 24 and 48, integrate 24 and 48 flow tanks, respectively. With the booming numbers of flow tanks compared to MinION, the PromethION system could output up to 7.6 Tb data while MinION could only generate 50 Gb within 72-h operation.

There are three forms of nanopore sequencing, 1D, 2D, and 1D^2^. 1D uses nanopore where only one strand of DNA is sequenced. 2D kit was first utilized in ONT. A hairpin structure was used at one end of the double-stranded DNA to connect two strands. After completing the sequencing of one strand, the sequencing of the other strand begins immediately. In this way, it is equivalent to repeat the sequencing twice, which can be used for base correction. 1D^2^ is similar to 2D, whereas it doesn’t need hairpins to physically bind the two strands of DNA together.

The reaction system for nanopore sequencing is carried out in a flow cell, in which two ionic solution-filled compartments were separated by membranes containing either 2048 (MinION) or 12,000 (PromethION) nanopores. The process of nanopore gene sequencing can be divided into three parts, library preparation, sequencing process, and basecaller.

### 3.1. Library Preparation

The preparation of the library is crucial for the subsequent work of nanopore sequencing. The DNA fragments should be repaired whether it has been sheared or not. When repairing, the repaired connector is a DNA-protein complex with a polymerase or helicase on the complex.

### 3.2. Sequencing Process

**Figure 2:**
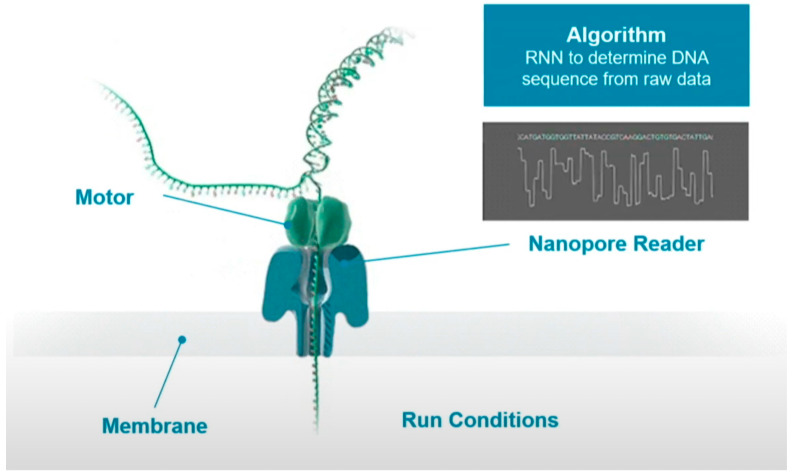
The process of nanopore sequencing [38].

Figure 2 shows the schematic process of sequencing. The DNA strand to be sequenced is mixed with copies of the processive enzyme. When the DNA-protein complex approaches the nanopore, the enzyme binds to a single stranded leader at the end of the double stranded DNA template, unzips the double strand, and feeds a single strand through the nanopore. A single molecule with high specificity can interfere with the current when the unzipped DNA long strand passing through the nanopore one base at a time. These current signals can be used to determine the type of base.

### 3.3. Basecaller

In the process of base readout, owing to the difference in the charge and structure of nucleotides when they translocate through the nanopore, the measured current would cause small disturbance. These electrical signals can then be translated into DNA sequences with the deep learning algorithms. However, the readout signals are noisy and random as these signals are originated from more than one molecule in the nanopores, which is difficult for the basecaller.

In addition, the resistance of the hole is determined by the bases of multiple nucleotides located at the narrowest point of the hole [39]. Being the last step for the interpretation of the entire DNA sequence, the data analysis using deep learning is challenging, which requires efficient algorithms and large amount of data for computational training.

## 4. Optimization Ways in ONT

Traditional nanopore sequencing technology is not able to replace the previous generation of sequencing devices owing to the high error rates, up to 5–15% [40,41]. To improve the sequencing accuracy of nanopore sequencing technology, it is necessary to analyze from the perspective of error source. Firstly, the Signal Noise Ratio (SNR) is affected by the inherent characteristics, including the structural similarity of nucleotides, the constant signal in the homopolymer, and the non-uniform velocity of nucleotide through the pore. The SNR needs to be optimized during the preparation of DNA library. Moreover, the process of translating the read current signal into base sequence is limited by the type of basecaller program, which needs to be optimized in the software and algorithms.

Also, the structure of nanopore structure can be upgraded to enhance the performance of the sequencing technology [42]. In 1D sequencing mode, R9 nanopore with 1 reader head can achieve the accuracy of 95% and consistent accuracy of Q44 (99.996%). However, one reader head cannot accurately identify base with multiple identical bases in a row. Hence, besides combining with the latest basecalling software, the number of reader head could increase to be dual headed. Also, the combination of the chips with one and dual reader heads can obtain higher consistent accuracy. In this review, the detailed ways for the optimization of nanopore sequencing are listed as follows:

### 4.1. Optimization in Library Preparation

This review mainly reports an optimization method called intramolecular-ligated nanopore consensus sequencing (INC-Seq), developed by the Singapore Genome Research Institute [43]. Based on the concept of PacBio’s CCS, the INC-seq repeatedly sequences the same molecule and hence a consensus sequence can be calculated to improve accuracy [44].

**Figure 3:**
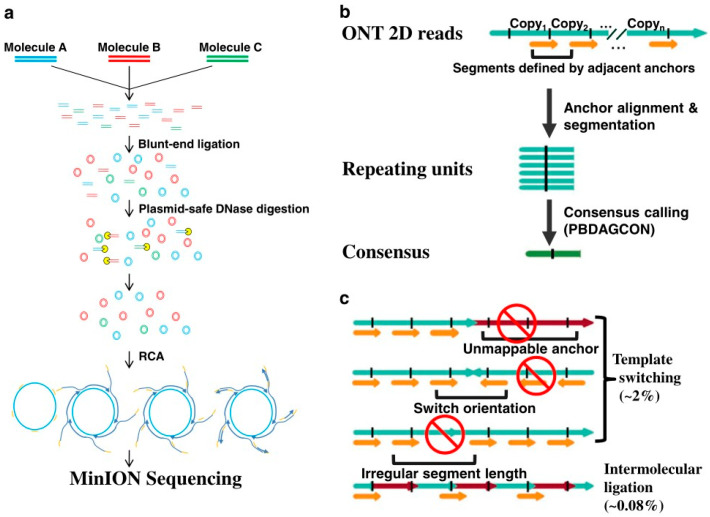
The schematics of INC-seq sequencing technology show (**a**) the process of making RCA, (**b**) the process of locating the repeat sequence with anchor, and (**c**) the sources of chimeras [43].

As the linear DNA template is needed in nanopore sequencing, the researchers adopted a novel library preparation scheme. As shown in Figure 3a, the template DNA molecule is first cycled and then amplified using roll-circle amplification to generate circular molecules. The circular molecules consist of multiple repeating units, which are then broken into linear DNA strands before sequencing on a nanopore sequencing platform.

After obtaining the rolling-circle DNA sequencing results, the sub-read from the original nanopore is used as anchors to scan the entire reading to find the location of duplicated units. The sequence results can be read from the consensus sequence by comparing between the start point and the end point of an adjacent anchor. In addition, in the process of library preparation, each read in INC-Seq is required to be tandem repeats of the same template for the calibration protocol, as shown in Figure 3b.

The main problem faced by this method in library preparation is chimerism, which can lead to a poor or chimeric consensus during sequence interpretation. As shown in Figure 3c, two types of patterns are exhibited in the chimeras of genes, one from template switching and the other from intermolecular ligations. The one from template could induce incompatible mappings of anchor points, such as unmappable anchor points and orientation switch, or irregular anchor distance.

To solve these two problems, researchers designed a bioinformatics pipeline that extracts repeating sequence segments and corrects these segments by constructing a consensus sequence, also called anchor-based consensus construction. Instead of detecting the chimeras from intermolecular ligations during the transmission process, the detection is made by detecting whether the consensus sequences are more than twice as long as expected. In the conditions of the INC-Seq experiment, the observation of chimeras from intermolecular ligations was very rare, which is only about 0.08%.

The INC-seq enables accurate species-level classification and identification at 0.1% abundance. It could robustly quantify the relative abundances on the MinION system. Moreover, by applying INC-Seq for 16S rRNA-based bacterial profiling, it could generate full-length amplicon sequences with a median accuracy >97% [43].

### 4.2. Optimization of Basecalling Programs

With the development of GPU, the traditional machine learning method, Hidden Markov models, has been replaced by deep learning methods, which enables to train more complicated neural network. Many different aspects could affect the predicted results, by the neural network, including the construction method of hidden layer, the architecture of the neural network for modeling, the species of biological genome corresponding to the data set in the training process, etc. [39]. Table 2 concludes the normal machine learning algorithms and deep learning networks that could be used to construct the training program. In addition, larger neural networks can improve the accuracy of basecalling and consensus sequences at the expense of sequencing speed [39].

**Figure 4:**
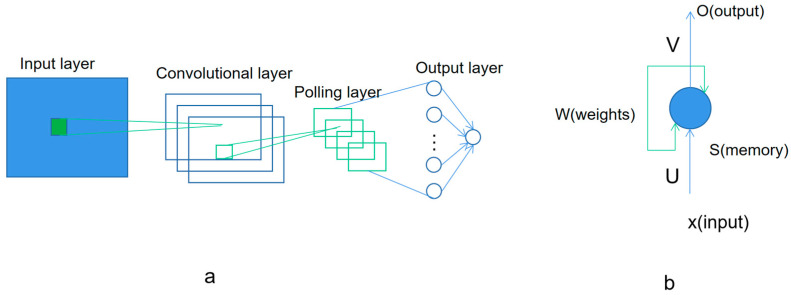
The base model of (**a**) the convolutional neural network and (**b**) the recurrent neural networks.

Figure 4 shows the base model of convolutional neural networks and recurrent neural network. The convolutional neural network (ConvNet), including CNN and CTC, has the ability to extract the features in the input training data. The pre-processing required in a ConvNet is much less compared to other classifications. With enough training, ConvNets is able to learn different features. The recurrent neural network, including RNN and LSTM, is another kind of artificial neural network where the connections between nodes in the network form a directed graph along a temporal sequence. This allows it to exhibit a temporal dynamic behavior. Deriving from feed forward neural networks, the recurrent neural networks can use their internal state (memory) to process inputs with variable length sequences [50].

Many studies have focused on improving the basecaller by developing an appropriate algorithm. When the MinION sequencing platform was launched in 2014, the basecaller was called Metrichor, a cloud-based EPI2ME platform that uses Hidden Markov models for basecalling. However, this service was discontinued in March 2017. In August 2016, the Minknow software began supporting basecalling, which can be downloaded to a PC for monitoring and controlling MinION sequencing. ONT also offers several other basecalling programs, including the command-line basecalling program Albacore, the programs naming Nanonet and Scrappie for research and testing [41]. In addition, many researchers have also developed several basecalling programs based on various deep learning algorithms, including Nanocall [51], SACall [52], DeepNano [53], Chiron [54], and BasecRAWller [55]. Nanocall is the first open basecalling source that could be downloaded free and operated offline. The Nanocall and SACall are reviewed as follows.

#### 4.2.1. Nanocall Basecalling Program

Nanocall is a program realizing deep learning algorithms using C when Metrichor was still servicing [51]. The advantage of Nanocall is that it offers offline operation when running basecalling program while Metrichor must be connected to the internet. Also, Nanocall uses Expectation maximization (EM) algorithm to perform several rounds to identify bases more accurately.

However, Nanocall has disadvantages compared to Metrichor, as it performs basecalling in the 1D form and, thus, it cannot integrate information from complementary chains in the 2D sequencing form. When it comes to 2D sequencing form, it uses a special signal when the hairpin structure passes through the nanopore to realize the separation of the two chains. The signal cannot be integrated due to the lack of base in the DNA backbone.

#### 4.2.2. SACall Basecalling Program

SACall is an end-to-end basecaller composed of the convolution layer, transformer self-attention layer, and a CTC decoder. It is a program to convert the current signal to base sequence by using neural network. In SACall, the convolutional layer is used to subsample the signal to capture the local information. To realize the upstream and downstream correlation of signals, the self-concern layer is used to calculate the similarity of every two positions in the original signal sequence. The CTC decoder uses a beam search algorithm to obtain the base sequence.

Huang et al. used a benchmark set consisting of nine independent genomes to compare the performance of SACall with the official test programs, including Albacore and Guppy, for nanopore sequencing [52]. SACall performs better than the other two programs in the readout accuracy, assembly quality, and consensus accuracy. SACall is also an open-source program with the advantage of wider spreading among researchers.

### 4.3. Optimization of Nanopore Types

Currently, nanopore R9.4.1 has been used in multiple areas for rapid sequencing and showed a good performance, including cancer research [56], human genetics [57] and microbiology [58]. However, as R9.4.1 has only one reader head, it cannot accurately identify bases in some special sequences. For example, in the homopolymeric regions with multiple continuous identical bases, the deletion of bases would occur when using R9.4.1 with one reader head.

**Figure 5:**
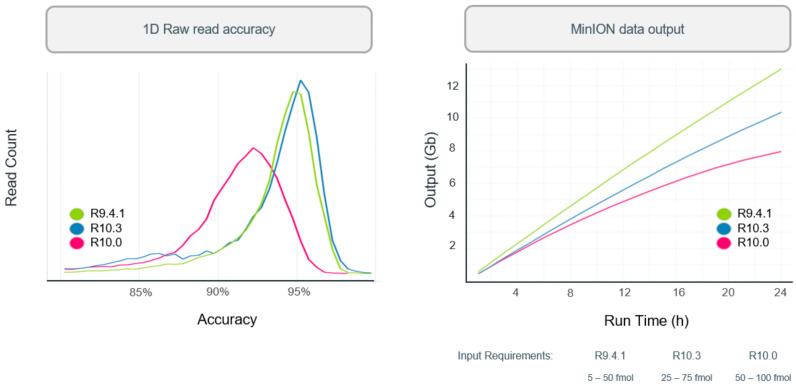
The comparison of performance using different types of nanopore. Oxford Nanopore Technologies Inc., Oxford, UK, 2020 [59].

In 2019, R10 was introduced with dual reader head, which can better recognize and correct the results when detecting DNA with identical bases. When DNA molecules pass through the nanopores of R10 chip, the dual reader would detect the sequence twice as high as that of R9 to determine the bases. Hence, it could better identify low complexity sequences, minimize errors, and improve consistent accuracy. Since then, the Oxford Nanopore R&D teams have been working to tune the nanopore to increase the throughput and accuracy. Similar to R10, R10.3 was introduced with dual reader header while the input amounts and raw accuracy were improved. The improvement of read accuracy and processing speed can be found in Figure 5.

## 5. Applications of Nanopore Technology

There are many applications using nanopore sequencing as it enables an easier and more accessible study of genetic and epigenetic modification as well as their role in gene expression [60]. Besides, in the area of biomedical research, nanopore sequencing can provide insights into the mechanism of diseases and solutions to the problems in medical diagnosis and testing from the perspective of gene and chromosome. For detecting the new types of virus and bacteria, nanopore gene sequencing also gives a high sequencing solution and a portable sequencing platform. This review will give representative applications of nanopore technology. Table 3 concludes the applications of the nanopore technology and some of the applications are mentioned particularly in the following part.

### 5.1. Diagnosis of Cancer by Gene Modification

#### 5.1.1. Structure Variations

The structural variation in the cancer genome is characterized by a large number of deletions, amplification, inversion, duplication, and translocations. Changes in these sequences usually lead to abnormality in gene regulation such as fusion genes and copy number changes, which may lead to activation or overexpression of oncogenes and inactivation of tumor suppressor genes [75]. Nanopore sequencing technology can overcome the structural variation challenges of short read long sequencing meets when it comes to copy with the long-repeated fragments.

Norris et al. detected the inactivation of tumor suppressor genes (CDKN2A/p16 and Smad4/DPC4) due to structural variation in pancreatic cancer using nanopore sequencing [76]. It shows that nanopore sequencing technology has a certain application prospect in the detection of a series of structural variations with good characteristics. Williams et al. also demonstrated that targeted nanopore sequencing is an effective way to identify ABCB1 structural variation in THP-1AML cells and HGSCs [63].

Nanopore sequencing gives access to genomic regions that may be inaccessible to traditional methods of sequencing. If nanopore technology can be applied in clinical practice, it will become an ideal tool for identification of structural variation and the early diagnosis, treatment, prognosis of cancer, and therapeutic monitoring.

#### 5.1.2. Transcription Factor

Transcription factor (TF) is the main regulation factor of gene expression and signaling pathway in all known biological systems. It is a group of genes that can bind specifically with specific sequences of the upstream 5’- terminal of the gene, and, thus, bind with specific DNA sequences to promote or suppress gene expression.

In cells, the majority of oncogenes and tumor suppressor genes encode TF [65,77]. Its abnormal activity can be used to characterize the presence of cancer clinically. Squires et al. used nanopores to analyze the ion current sublevels generated by the translocation of the ZIF268/DNA complex by single molecular analysis, proving that nanopore technology can be used to distinguish the specific and non-specific binding conformations of ZIF268 [78]. This further demonstrated nanopore to be capable of detecting complex structures and protein conformations.

These studies open the door to new applications of nanopore technology, which can be used to study DNA complex research work and is expected to be targeted to guide the diagnosis and treatment of cancer. Furthermore, nanopore sequencing technology also gives a chance to investigate molecular mechanisms of the TFs [79]. Also, it could provide an in-depth insight into a broad panel of human cell lines [80].

#### 5.1.3. Telomeres

Telomeres are DNA-protein complexes at the ends of linear chromosomes in eukaryotic cells, which forms a special “cap” structure together with telomere binding proteins. Telomeres, centromeres, and replication origin are the three essential factors for chromosome integrity and stability. Telomeres shorten by an average of 19 bases per year due to aging, oxidation, stress, mitotic activity, and lifestyle habits.

Ding et al. used α-hemolysin as a nanopore sensor to study the folding structure of I-motif in human telomeres at different pH values [81]. The potential of α-hemolysin as part of biosensor development has been demonstrated. It is helpful for us to understand the lifetime and biologically related structure of I-motif of telomere sequences.

If the nanopore technology is proved to be valid to investigate the telomeres, we can better understand the dynamics and mechanisms of telomeres and analyze the relationship between telomere length and environment factors, including oxidation, stress, etc. In addition, the study of telomere G-quadruplexes molecules in the telomere structure of cancer cells is expected to provide guidance for the diagnosis and treatment of cancer in a broader way [82,83]. By studying the telomeres, more binding molecules on G-quadruplexes may be explored to be applicable to the treatment of a wide range of human cancers.

### 5.2. Diagnosis of Cancer by Epigenetics

#### 5.2.1. DNA Methylation

In the human genome, DNA methylation is an epigenetic modification, including 5-methylcytosine (5-mC), N4-methylcytosine (4-mC), and N6-methyladenine (6-mA) [84]. In mammalian cells, CpG methylation can directly or indirectly suppress gene expression [85]. Moreover, the degree of abnormal methylation (high or low methylation) is associated with some cancers. DNA methylation pattern is one of the earliest and most common molecular changes in human tumors [86]. Studies have shown that almost all cancer types have hundreds of genes with an abnormally increased methylation [87,88]. There is also an increasing number of studies using nanopore sequencing technology to further investigate DNA methylation.

Jiwook et al. [89] proposed a nanopore array method for DNA methylation detection, which bypassed traditional bisulfite conversion [90] and amplification by PCR reaction [91], demonstrating that the use of 10 nm nanopore can distinguish between hypermethylated and unmethylated dsDNA oligonucleotides. The work provides a direct electrical analysis technique to detect various methylation levels on DNA fragments at the single-molecule level, showing the potential of nanopore to identify abnormally methylated DNA in clinical tests aimed at diagnosis of diseases such as cancer.

Lee et al. [60] used nanopore sequencing technology to detect endogenous CpG methylation and at the same time to exogenously label chromatin accessibility sites. By analyzing four human cell lines, the team constructed a human epigenome map that included information on CpG methylation and chromatin accessibility, and revealed differences in methylation and chromatin accessibility between breast cancer cells and non-cancer cells.

The research mentioned above both demonstrated that the use of nanopore technology to detect abnormal DNA methylation may play an important role in cancer therapy and precancerous detection [92,93]. The nanopore-based methylation-sensitive assay provides a more convenient method in studying the role of epigenetics in human disease without bisulfite conversion, fluorescent labeling, and PCR.

#### 5.2.2. MicroRNA

MicroRNAs (miRNAs) are small endogenous biomolecules, with a length of 18–22 bps, which can regulate gene expression and their expression level is correlated with different diseases [94]. Studies have shown that abnormal expression of miRNAs has been found in different tumor tissues [95,96]. In addition, miRNAs play important roles in embryonic differentiation, hematopoiesis, cardiac hypertrophy, and many cancer-related processes, including proliferation, apoptosis, differentiation, migration, and metabolism [97,98].

Many research groups have used biological and solid-state nanopores to detect miRNAs in different tissues. For example, Meni et al. [99] used nanopore technology for rapid detection of probe-specific miRNAs (miRNA-122a and miRNA-153) and proved the potential of this approach by detecting liver-specific miRNAs at microgram levels from rat liver. Wang et al. [100] selectively detected single-molecule miRNAs in plasma samples of lung cancer patients using α-hemolysin-based nanopore sensors. The sensor uses programmable oligonucleotide probes to generate target-specific signals that quantify sub-millimolar levels of cancer-associated miRNAs.

These methods have potential application value for quantitative miRNA detection, detection of disease markers, and non-invasive early diagnosis of cancer. Also, nanopore gene sequencing method enables researchers to investigate overexpression mechanism of many kinds of miRNA.

### 5.3. Detection of Viruses and Bacteria

#### 5.3.1. Monitor Virus Using Nanopore Sequencing Technology

During a pandemic, gene sequencing is an ideal way to determine the source of infection and the rate at which it evolves. Gene sequencing technology can also be used to identify characteristics of host fitness, identify and detect diagnostic targets, and characterize responses to vaccines and treatments [67,101].

Human beings’ struggling against viruses is an evolving process. In the face of Ebola virus in West Africa, some scientists proposed to use nanopore sequencing technology to quickly detect virus samples in the environment, so as to help identify virus mutation types. The sequencing device, MinION, was used. In 2016, the Zika virus outbreak was declared an international public health emergency by the World Health Organization. The Zika in Brazil Real-time Analysis project was established to sequence a thousand of genomes from Brazil to monitor the epidemiological information [102]. The whole genome of Zika virus was obtained from clinical samples using the MinION sequencer for bioinformatics analysis [40]. MinION was used under these circumstances as it has many advantages. It weighs less than 100 g and is easy to operate in developing countries where the experimental condition cannot be achieved. Another advantage is that nanopore sequencing does not require precise microscope alignment and repeated calibration in comparison to the Illumina platform [103] and, hence, make the operation of MinION much simpler.

In view of the ongoing global epidemic of the 2019-nCoV virus, many teams used nanopore sequencing technology to explore and detect the novel coronavirus [104,105,106]. Among them, Wang et al. [105] proposed a nanopore targeted sequencing (NTS) technology to detect SARS-CoV-2 and other respiratory viruses simultaneously within 6–10 h. Researchers proved that NTS can effectively monitor the mutation of nucleic acid sequences, categorize the type of SARS-CoV-2 and other respiratory viruses in the samples.

In the future, with the rapid sequencing devices of nanopore sequencing, it is promising to establish virus surveillance and further establish rapid responsive system to track and monitor the diseases that threaten public health.

#### 5.3.2. Study on Bacteria Using Nanopore Sequencing Technology

Nanopore technology could also be used for investigating the bacteria. The traditional method for bacteria research is to study the 16S rRNA of bacteria [107]. Brandt et al. [68] provide a promising method for monitoring the abundance of community present in microbial anaerobic bacterial communities by using metagenomic reading classification of nanopores, which can be used as an alternative method for bacterial taxonomy using 16S rRNA.

In recent years, the antibiotic resistance of bacteria has become a threat to aquatic and terrestrial biodiversity [108]. Also, it is crucial to investigate the resistance gene in bacteria with the gene sequencing method. Zhang et al. [109] used Nanopore sequencing platform and Illumina sequencing platform to quantitatively study the major antibiotic resistance gene types, showing that nanopore sequencing technology could be used for quantitative study of bacterial resistance. The result is conducive to promoting the research on antibiotic resistance gene transfer in sewage treatment plants and promoting the solution of the hot issue of antibiotic failure [110].

### 5.4. Assembly of Genome

Due to the characteristics of high throughput and long read-length, nanopore sequencing technology has more advantages in assembling genomes compared to the traditional short-read-long method. It can produce a more continuous genome assembly in the process of whole-genome assembly by crossing highly repetitive regions and regions containing structural variation [111,112].

**Figure 6:**
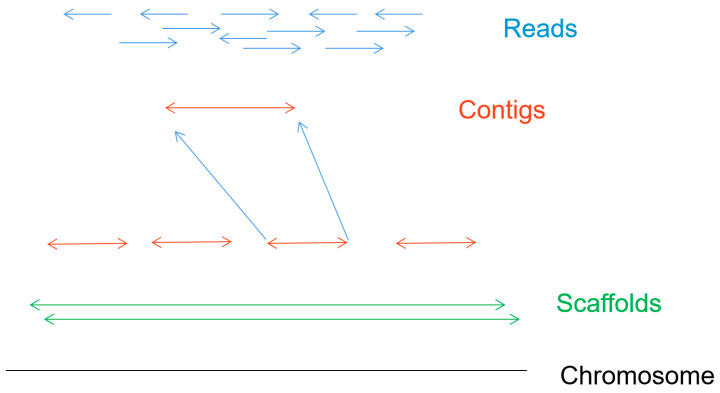
The process of assembly of chromosomes.

Figure 6 shows the de novo assembly of chromosomes, which is the process of determining the order of each base in the genome by reconstructing randomly sampled sequence fragments [6]. The first step is to get sequence fragments (reads) by using the nanopore gene sequencing device. Splicing the short read according to the overlap between reads, the longer continuous sequences (contigs) are formed. Then the scaffold can be formed by joining the contigs together. After eliminating the error of scaffolds and gaps in the scaffold, a high-quality whole genome sequence can be obtained.

Readings from the same region of the genome can be linked together to form overlapping clusters. When using short-read sequencing technology, the repeated lengths exceeded the overlapping lengths can cause ambiguous reconstructions and fragmentation of assembly fragments. In comparison, nanopore sequencing technology can solve this problem by taking advantage of the ultra-long reading length.

To assemble the diploid genomes, such as human genomes, the challenge is to achieve accurate haplotype solutions from telomere to telomere without reference. Many studies have pointed out that short-read sequencing technology is insufficient to traverse most of the repeating structures of the genome [113]. In comparison, nanopore sequencing technology is promising with the ultra-long reading length [6,114]. An example is the sequenced genomes in GenBank. At the beginning of 2015, the major genome data was mainly achieved by short read gene sequencing technique, with only 99 mammalian genome combinations. The average overlap group N50 was only 41 kb [115]. Benefiting from the long-read sequencing technology such as PacBio and ONT by the year 2020, the average overlap group N50, whose length is more than 800 genomes, in the GenBank was greater than 5 Mb [23].

## 6. Conclusions

Detecting the potential change when charged biological molecules smaller than nanopores pass through the hole, nanopore technology provides a new way to identify and quantify a variety of analytes. Nanopore sequencing technology combines nanopore technology and biosensors, and it will have a tremendous impact in the area of gene sequencing.

Nanopore sequencing technology has broad application prospects in gene modification, epigenetics research, detecting microbes, and other related fields. It also plays an increasingly important role in human cancer diagnosis and other medical tests. Also, if the problem of high error rate can be solved and the ONT devices can reach the same level of accuracy, fourth-generation sequencing technology can take the place of the second-generation technology, which will dominate the market in the near future. In the future, nanopore sequencing technology will be continuously optimized from the structure of nanopore, machine algorithm of basecalling, and new methods of library preparation. It will lead human beings to continuously explore new types of gene variation and help people better solve the problems encountered in modern medicine.

At the same time, in the field of life science research, more reference genomes will be constructed, nanopore technology will be embedded into more kinds of devices and further broaden the understanding of genetics, epigenetics and transcription, variation, and its relationship to human phenotypes.

## Figures and Tables

**Table 1:** The comparison of different generation of gene sequencing.

	Reading Length (kb) N50	Estimated Cost per Gb (US $)	Throughput per Flow Cell (Gb)	Read Accuracy (%)
Sanger(1st)	<1 kb	13,000 ^d^	/	>99.9
Illumina(2nd)	0.075–0.15 ^a^	50–63	16–30	>99.9
PacBio(3rd)	10–20 ^b^	43–86	15–30	>99
ONT(4th)	10–60 ^c^	21–42	50–100	87–98

The estimated cost in a, b, c excludes the cost for labor, instrumentation, maintenance, and computer resources. ^a^ Illumina NovaSeq 550 works in single-end mode. ^b^ PacBio Sequel II works in HiFi mode. ^c^ ONT PromethION works in long mode. ^d^ The cost was calculated by the HCV genome from [21] and the cost includes the operating costs. Data in the table comes from [21,22,23].

**Table 2:** Neural network algorithms used in nanopore basecaller.

Algorithms	Descriptions
Hidden Markov models [45]	A stochastic model that makes predictions based only on the previous event and a series of observations.
Recurrent Neural Network (RNN) [46]	A model that allows networks with periodic connections to learn complex tasks that require information to be maintained for fixed or indeterminate periods of time.
Long-short-term memory (LSTM) [47]	A type of recurrent neural network that can be used as a component of a larger network. It has specific input, output, and forgetting gates, which can be implemented to retain or discard information passed from the previous state.
Convolutional Neural Network (CNN) [48]	A neural network model for image classification, which extracts input features through a convolution algorithm.
Connectionist Temporal Classification (CTC) [49]	A convolutional neural network for marking neural network output and scoring of sequence data. It does not require pre-segmented training data and post-processed output

**Table 3:** Applications of Nanopore Technology.

Applications	Descriptions
Clinical research	With nanopore technology, the long read can help researchers to identify and phase genetic variant, and fully characterize novel isoforms and fusion transcripts. Nanopore technology gives a new insight to health and disease, from cancer, immunology, to neuroscience. The representing references are [61,62,63,64,65,66]
Detection of microbes	The nanopore technology can be used to sequence the DNA or RNA sequence of microbes, and it helps researchers to classify or monitor the microbes. Moreover, it is promising to establish microbe surveillance and response quickly during pandemic, if the nanopore technology can be applied in the area of public health. The representing references are [34,35,67,68]
Assemble genomes	Owing to the long-read sequencing ability, nanopore sequencing technology can overcome the problems that short-read sequence devices meet in area the long-repeated fragments. The representing references are [35,69,70,71]
Environmental genomics	The portable and affordable nanopore sequencing technology provides a unique tool for environmental research, including biodiversity assessment, pathogen identification and animal conservation. Besides, the real-time data analysis provides immediate access to results, whether in labs or in the field. The representing references are [72,73,74]

## Data Availability

Data is contained within the article.
